# A high throughput synthetic workflow for solid state synthesis of oxides†

**DOI:** 10.1039/d3sc05688k

**Published:** 2024-01-02

**Authors:** Christopher J. Hampson, Moli P. Smith, Luca L. Arciero, Christopher M. Collins, Luke M. Daniels, Troy D. Manning, Michael W. Gaultois, John B. Claridge, Matthew J. Rosseinsky

**Affiliations:** a Department of Chemistry, University of Liverpool, Materials Innovation Factory 51 Oxford Street Liverpool L7 3NY UK M.J.Rosseinsky@liverpool.ac.uk

## Abstract

High-throughput synthetic methods are well-established for chemistries involving liquid- or vapour-phase reagents and have been harnessed to prepare arrays of inorganic materials. The versatile but labour-intensive sub-solidus reaction pathway that is the backbone of the functional and electroceramics materials industries has proved more challenging to automate because of the use of solid-state reagents. We present a high-throughput sub-solidus synthesis workflow that permits rapid screening of oxide chemical space that will accelerate materials discovery by enabling simultaneous expansion of explored compositions and synthetic conditions. This increases throughput by using manual steps where actions are undertaken on multiple, rather than individual, samples which are then further combined with researcher-hands-free automated processes. We exemplify this by extending the BaY_*x*_Sn_1−*x*_O_3−*x*/2_ solid solution beyond the reported limit to a previously unreported composition and by exploring the Nb–Al–P–O composition space showing the applicability of the workflow to polyanion-based compositions beyond oxides.

## Abstract 2

A slurry based solid-state high-throughput workflow provides sample forms suitable for presenting to automated characterisation for the discovery of new oxide materials.

## Graphical abstract

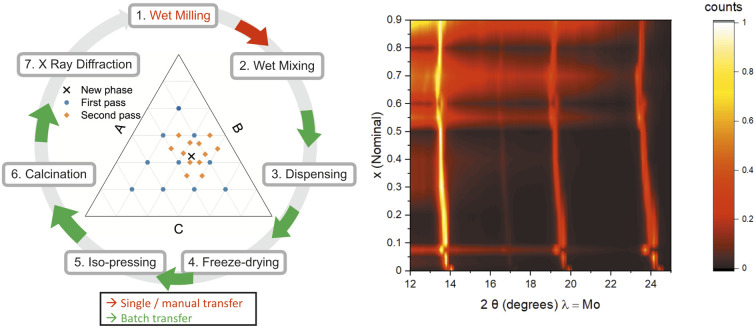

Traditionally the sub-solidus synthesis method of mixing dry raw materials followed by repeated cycles of hand grinding and calcination has played a central role in the discovery of new materials by solid state chemists because of its versatility in terms of accessible chemistry and applicable reaction conditions.^1^ The method is labour intensive and only processes one formulation at a time.^2^ In the ceramic industry development often uses a small-scale version of the industrial process, usually wet milling followed by drying, granulation, compaction to a desired shape followed by high temperature sintering. As the pace of technological change increases and the need for higher-performance solid-state materials for batteries, solar cells, and catalysts becomes ever more urgent, these tried and tested methods are no longer adequate to explore the large chemical and reaction parameter space.^3^ The increasing number of elements involved in materials used as dielectrics, piezoelectrics and in other functional materials serves to exacerbate the problem as the compositional space to be explored grows exponentially.^4^

The use of high throughput parallel processing and testing in the pharmaceutical industry is well established,^5^ and has extended to other areas where liquid formulations are required such as personal care and cleaning products.^6^ It has generated a range of automated liquid handling techniques from laboratory bench to industrial scale. However, the development of high throughput methods for solid materials is more limited and has tended to focus on thin film deposition technologies.^7–10^ Libraries of materials have also been produced as spots on substrates^11^ and by ink-jet printing^12,13^ but the amounts of material produced are very small (mg scale) and the applicability is limited by problems of reaction with the substrate during calcination and of the substrate appearing in the subsequent X-ray diffraction analysis. Sol–gel based methods, when this synthesis chemistry is appropriate for the system under study, are ideally suited to the high-throughput liquid dispensing robots developed for the pharmaceutical industry and have been applied to the discovery of new battery electrolyte materials,^14,15^ metal halide perovskites^16^ and work has been done to introduce solid handling into a high-throughput ceramic workflow.^17^ Continuous hydrothermal flow synthesis has also been used to prepare libraries of powdered oxides for oxygen evolution reaction electrocatalysts, producing individual sample sizes of ∼3 g.^18^

Traditional solid-state synthesis is challenging to achieve in a high throughput workflow, as the parallelisation of mixing, grinding and pressing many 10's of solid samples demands bespoke solutions^19–21^ or multiple milling tools for avoidance of media contamination. Alternative workflows that are complementary in terms of accessible chemistries, reaction conditions and sample form factor to those already established will expand the range of investigations that can be accelerated through automation. In this work we have combined elements of ceramic processing with automated liquid handling and other process steps from pharmaceuticals to develop a high-throughput route for solid materials development which delivers larger quantities of material (typically 100–250 mg) in free-standing samples suitable for a range of subsequent measurements. Initially the route has been used to generate a range of oxide materials as part of a programme to screen ternary and higher compositions for previously unknown multi-component phases by X-ray diffraction.

The overall concept of the workflow that we present here is illustrated in Fig. 1a. Precursor powders are mixed as aqueous slurries using robotic liquid dispensing into sacrificial well plates to form discrete solid pellets (after drying, compaction and calcination steps) which can be presented to automated instrumentation for characterisation. The overall effect is to increase throughput by automating some steps and modifying others so that manual actions now impact multiple samples, particularly during transfer between processes. This reduces the researcher time needed to achieve an equivalent volume of data, or enables richer data to be generated with equivalent effort. Particular focus has been given to the elimination of manual “one by one” operations and reducing handling to sets of multiple samples wherever possible. The retention of some manual handling and therefore inspection of the samples at intermediate points in the process gives flexibility and resilience, so that subsequent choices can be adjusted (*e.g.*, calcination temperature or reaction atmospheres). In a research project whose purpose is the discovery of previously unknown materials and structures, a totally automated process may not be appropriate or proportionate, with the retention of manual steps adding flexibility for intervention to make changes based on learning, while automation is used to accelerate steps that are more readily engineered. The aim of the workflow set out here is discovery by screening, with precise measurement of individual hit compositions required in subsequent serial studies.

**Fig. 1:**
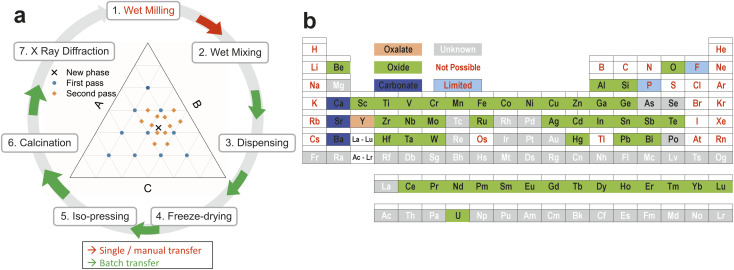
Slurry based high throughput workflow and components (a) overview of the high-throughput ceramic synthesis workflow with the detail behind each step 1–7 provided in the Methods section. Milling of aqueous precursor slurries allows liquid handling robots to dispense and mix sets of mixtures that are then dried, compressed and calcined to enable presentation of discrete pellets of dense powder samples to X-ray diffraction or other forms of characterisation. (b) The range of elements that can be used in the slurry based high-throughput workflow we describe for materials discovery or targeted materials synthesis.

## 1 Experimental

The individual workflow steps numbered in Fig. 1 are described in more detail below.

### 1.1 Wet milling

Insoluble raw materials (oxides, carbonates, oxalates) were milled in deionised water using zirconia media in a Fritsch Pulverisette 7 planetary mill. An ammonium polyacrylate dispersant was used to reduce suspension viscosity and a water-based acrylic emulsion binder was added to increase the mechanical strength of the discs after drying so that they could be isopressed to increase their density and would remain intact through subsequent calcination and embedding. Each mill generated 15 cm^3^ of aqueous suspension with a known content of inorganic precursor per unit volume. The solids contents were checked by drying a 1 cm^3^ sample taken from each mill in an oven at 80 °C overnight and this measured solids content was used to calculate the weight and molarity of inorganic precursor per unit volume of suspension and thus the volumes required in the later dispensing operation.

The suspensions were kept on a disc type sample rotator to prevent sedimentation/separation. A set of three suspensions allows a quaternary oxide system (A–B–C–O) to be addressed. With the current equipment a fourth component could be added and there is no reason in principle why the technique could not be expanded to more complex formulations.

The generation of these suspensions is a conventional process on a small scale which requires significant manual intervention and which is essentially unchanged from normal laboratory practice – hence it is shown as a manual process in Fig. 1a.

### 1.2 Wet mixing

Mixing of the aqueous suspensions was carried out using an Eppendorf epMotion 5075 automated liquid handling station. The starting material suspensions were held on a specially constructed low-profile multi-position magnetic stirrer so that the solids did not settle out during dispensing. The current version of the stirrer has places for four suspensions held in glass vials. The stirrer uses rotating magnetic fields from three sets of stationary coils which are directed to the four stations *via* soft iron pole pieces. The design minimises the height of the bottom of the vials to increase the volume of material that can be processed, currently to 30 cm^3^ per suspension. Stirring speed can be varied from 240–960 rpm. The conventional PTFE bar stirrers originally used have been replaced with a custom designed 3D printed stirrer with embedded neodymium iron boron magnets. The design has a centre hole which allows the Eppendorf dispensing tips to aspirate from a lower level and reduces the dead volume of liquid which cannot be taken from the vial. The components were dispensed by volume into glass vials (one per formulation) and then the liquid handler mixed each formulation by repeated aspiration and dispensing. The dispensing parameters of the Eppendorf handler were adjusted to allow for the increased density and viscosity of the suspensions compared to water and optimisation of these parameters should allow closer match between nominal and measured compositions.

### 1.3 Dispensing

**Fig. 2:**
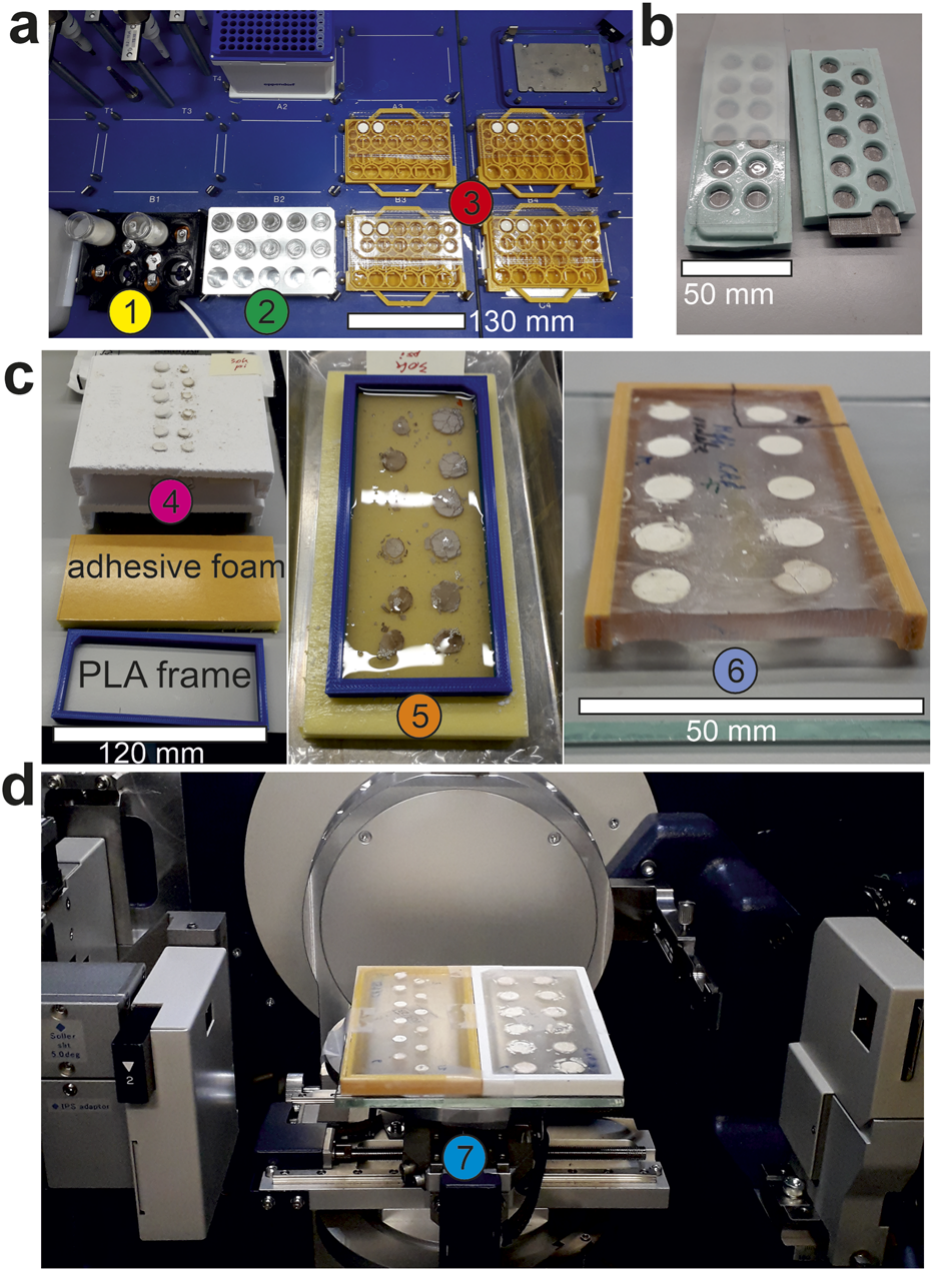
Slurry-based high throughput ceramic synthesis workflow (a) image of the robotic slurry dispensing components. (1) Low profile stirrer holding two precursor slurries for dispensing into a rack of vials (2) for mixing the precursor slurries in the required ratios, which are subsequently dispensed into (3) four sets of sample trays on 3D printed holders containing aliquots of the slurry mixtures; (b) isopress tooling showing an unfilled PET tray supported by a silicone holder. The metal insert ensures the rigidity of the holder and the flatness of the pellet faces. The silicone cover sheet protects the samples and prevents them falling from the PET tray during processing; (c) images of a sample set (4) positioned on refractory batts after calcination, a calcined sample set (5) on an adhesive polymer foam pad surrounded by a PLA frame and filled with epoxy, (6) cross-section of a cured epoxy resin block with embedded calcined sample set with flat surfaces uppermost, ready for placing onto an automated XY-stage (7) inside the X-ray diffractometer; (d) two sample sets, embedded in epoxy loaded onto the XY-stage of the X-ray diffractometer. Scale bars are added for reference.

Small aliquots (currently 0.2 cm^3^) of each mixture were dispensed into specially designed and manufactured vacuum-formed transparent PET trays (Fig. 2a and S1†). The PET is chosen and tested to ensure that no catalyst or filler residues are left when it is subsequently burnt away. Other polymers including biodegradable plant-based materials could be substituted^22^ provided that they can be vacuum formed and also leave no measurable inorganic residue after combustion (*e.g.*, https://fkur.com/en/applications/trays-from-bioplastics/). Moulds to make the trays were generated by 3D printing using eSun High Temperature resin [https://www.esun3d.com/high-temp-resin-product/] on a Creality LD-002H printer. Tray width is limited by the need to fit them into a laboratory isopress (see below). The initial tray design held 12 wells, each approximately 10 mm in diameter and holding 0.2 cm^3^ of suspension. Further development is in hand to reduce the well size and thereby increase the number of samples per tray. The trays were held on custom 3D printed holders to locate them in the Eppendorf unit, mimicking part of the layout of a standard 96-well plate. These holders also facilitate subsequent handling. Multiple arrays of compositions can easily be generated for processing under various conditions. For example, in Fig. 2a one set of compositions is being dispensed into four trays for calcination at different temperatures. The same approach can be used to address variations in other process parameters such as compaction pressure, calcination time, calcination atmosphere. From this point on the samples are always handled as sets – there is no one by one handling of individual sample discs.

### 1.4 Freeze drying

The trays, on their holders, were manually transferred to a freezer at −20 °C overnight and then to a Labconco vacuum cabinet attached to a freeze drier. The metal shelves of the cabinet were covered with open cell polymer foam to insulate the trays from the shelves and prevent thawing of the samples during drying. Each liquid aliquot dried to form a porous disc with a flat-bottomed face. This bottom face will subsequently be used for XRD analysis.

### 1.5 Isopressing

After drying, the trays of discs were supported in custom made silicone holders (Fig. 2b), covered in 2 mm silicone sheet and vacuum sealed in nylon bags (Fig. S2†) before iso-pressing in an Autoclave Engineers 75 mm diameter wet bag laboratory isopress at 15 000 to 30 000 p.s.i. (105–210 MPa) to increase the density and strength of the discs. The silicone holders contained metal inserts to improve the flatness of the bottom faces of the discs. The trays were then inverted onto refractory batts (Fig. 2c) so that the flat faces of the discs were uppermost thus avoiding reaction between this sample surface and the refractory.

### 1.6 Calcination

Calcination was carried out in electric box furnaces with a carefully controlled heating cycle which burnt away the PET trays leaving the discs in their original positions on the refractory tray (Fig. 2c). The key element of this cycle was to limit the heating rate to 25 °C h^−1^ between 250 °C and 500 °C in order to avoid movement of the discs during the combustion of the PET trays thus preserving the layout and spacing of the samples.

A key element of the process is that the PET trays have carried the samples as sets since they were mixed as suspensions, avoiding any handling of individual samples. As the method develops further and sample numbers increase this becomes ever more important.

### 1.7 X-ray diffraction

The sets of samples were lifted from the refractories using self-adhesive polymer foam (Technical Foam Services, Corby, Northants, foam grade TFS503 + 4210 SAB adhesive, Fig. 2c) so that the flat faces of the calcined discs were stuck to the foam. They were then surrounded by a 3D printed PLA frame (Fig. S3†) which was partially filled with epoxy resin to embed the discs (Fig. 2c). The frame ensures that the top face of the discs will sit parallel to the table in the X-ray diffractometer without requiring that the two faces of the cast epoxy be parallel or that the casting is done on a precisely level table (Fig. S4†). The one problem we have faced with this system is that the asymmetric filling of the frames can lead to bending as the resin completes its curing. Two solutions to this problem were identified. Curved blocks could be straightened by putting them on a flat metal plate and under a metal weight (>500 g) in an oven at 125 °C for 1 hour and then cooling with the plate and weight still in place. To avoid having to do this extra step, the hardener to resin ratio was reduced from 0.5 : 1 to 0.27 : 1 and the bowing of the frames was reduced to within 0.05 mm of flat (Fig. S5†). Further details are given in the ESI.†

The adhesive was released from the cured epoxy using acetone, leaving the samples with their flat surfaces exposed (Fig. 2). Powder X-ray diffraction measurements were carried out in reflection (Bragg–Brentano) mode using a Rigaku SmartLab instrument equipped with a 9 kW rotating anode source providing a parallel beam of Mo radiation Kα radiation (0.70930 and 0.71361 Å). The arrays of samples were fixed to an XY table (Fig. 2d) where individual sample positions were selected from an image taken using a camera and scanned in series automatically overnight. For the four samples shown in Fig. 4 “measured with LaB_6_”, samples were removed from the epoxy resin block and remaining resin removed by calcining the samples at 550 °C for 4 hours. The powder was then mixed with LaB_6_ (cubic lattice parameter = 4.156852(2) Å) in an approximate 1 : 1 ratio by mass, as an internal standard to confirm the measured lattice parameter. These samples were then measured on a Phillips PANalytical diffractometer with a monochromatic CoKα1 source (*λ* = 1.78901 Å) in Bragg–Brentano geometry. For all diffraction patterns, Pawley refinements were performed using TOPAS academic.^23^ With the exception of the cubic perovskite phase in BaY_*x*_Sn_1−*x*_O_3−*x*/2_ samples, which was allowed to freely refine, lattice parameters for known phases were initially fixed, and all instrumental parameters refined (background, peak shape and zero error), then lattice parameters were released to obtain the refined values.

Chemical analysis of the BaY_*x*_Sn_1−*x*_O_3−*x*/2_ samples was performed on a spare set of discs which had not been calcined. Samples from these (∼10 mg) were digested in Parr vessels at 200 °C for 8 hours using 5 cm^3^ of 38% HCl. The solutions were diluted to 50 cm^3^ giving concentrations of Ba, Y and Sn of the order of 10–100 ppm which were determined using ICP-OES.

## 2 Results and discussion

The slurry based, high throughput ceramic synthesis workflow described here allows us to explore formulations containing the elements shown in Fig. 1b. It is possible with this workflow to implement the majority of the elements in the periodic table, as oxides, carbonates or oxalates.

Magnesium and lanthanum present some processing challenges in aqueous systems as their suspensions are unstable and thicken during storage. Some phosphorus- and fluorine-containing systems can be addressed by the use of insoluble compounds. For example, Nb_2_P_4_O_15_ and AlPO_4_ can be used to address a large part of the Nb–Al–P–O system, which is described below. In principle the method could be adapted to work with suspensions in a non-aqueous solvent. This would extend the range of elements that could be addressed though the milling, dispensing and freeze-drying equipment would need to be adapted to process the required liquid safely.

The slurry dispensing apparatus is shown in Fig. 2a utilising a commercial Eppendorf liquid handling robot. With three components and a 0.2 cm^3^ individual sample volume this system can deliver up to 200 discs from a single set of suspensions. After isopressing, using the support tooling shown in Fig. 2b, and calcination a set of discs with flat faces are produced which, when embedded in epoxy resin (Fig. 2c), are suitable for presentation to an X-ray diffractometer with an automated XY-stage (Fig. 2d) for initial assessment of phase identification. This enables further measurements that can be carried out on an approximately flat surface such as Raman spectroscopy, approximate measurements of permittivity, resistivity/conductivity and SEM/EDX for imaging and compositional analysis. If more precise chemical analysis is required to confirm the composition of particularly interesting samples then they are cut out of the resin blocks and the resin is easily burnt off at 550 °C before digestion for ICP analysis. With this in mind an epoxy resin with no measurable ash content was chosen.

Initial trials to optimise the reproducibility of the wet dispensing step are described in the ESI.† Once all the processing steps were established, we used the complete process initially to produce a simple ternary compound, calcium titanate. We then used the complete process for more complex systems, to show that expected phases will form under the process conditions with the ultimate aim to identify previously unknown compositions and structures in the phase fields. This resulted in expanding the solid solution range of a previously reported quaternary oxide through the identification of an unreported composition.

### 2.1 Synthesis of CaTiO_3_

**Fig. 3:**
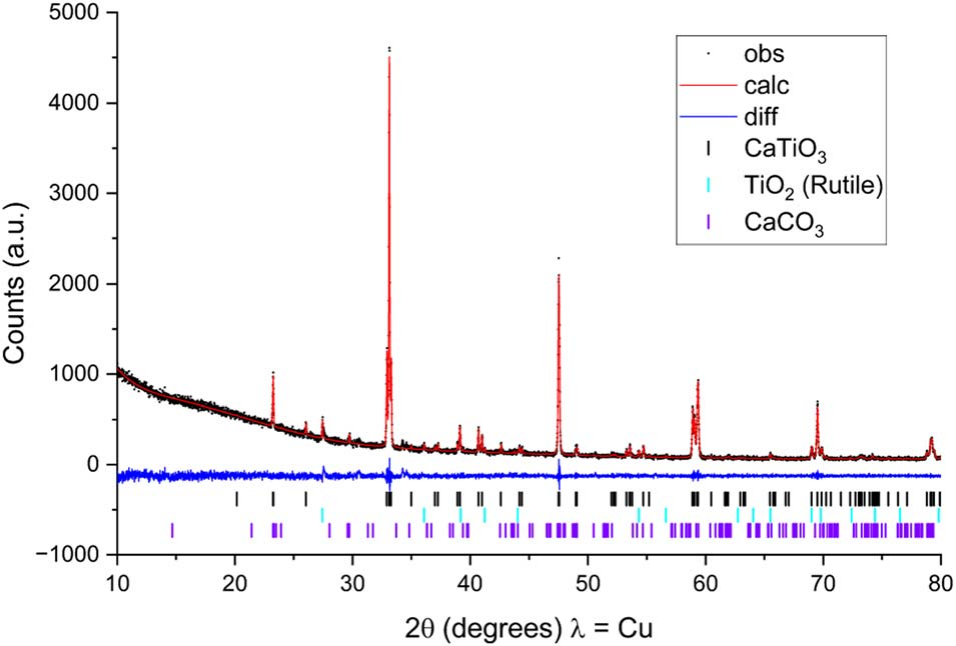
Powder X-ray diffraction pattern and Pawley fit of the reaction products from a mixture of CaCO_3_ and TiO_2_ slurries prepared following the described workflow. The majority product is CaTiO_3_ with some unreacted TiO_2_ and CaCO_3_ as impurities, confirming that the workflow is suitable for the desired aim of materials discovery.

As a first demonstration of the complete process and to show that carbonates could be used in this process route, a suspension of calcium carbonate was mixed with a titania (rutile) suspension in a 1 : 1 mol ratio and processed using the full workflow described above. Powder X-ray diffraction data obtained for a sample calcined to 1150 °C (Fig. 3) for 4 hours shows that the vast majority of the material has reacted to form calcium titanate with only a very small amount of unreacted rutile and calcium carbonate formed during cooling from residual CaO. This example shows that the initial steps provide adequate mixing and accurate dispensing of quantities (Fig. S6†) to enable successful calcination of the mixture leading to reaction to yield the desired product. The confirmation that the reagents used react successfully is critical as our main aim here is materials discovery where the formation of phases, either known or unknown, is required.

### 2.2 Exploration of the Ba–Y–Sn–O phase space

**Fig. 4:**
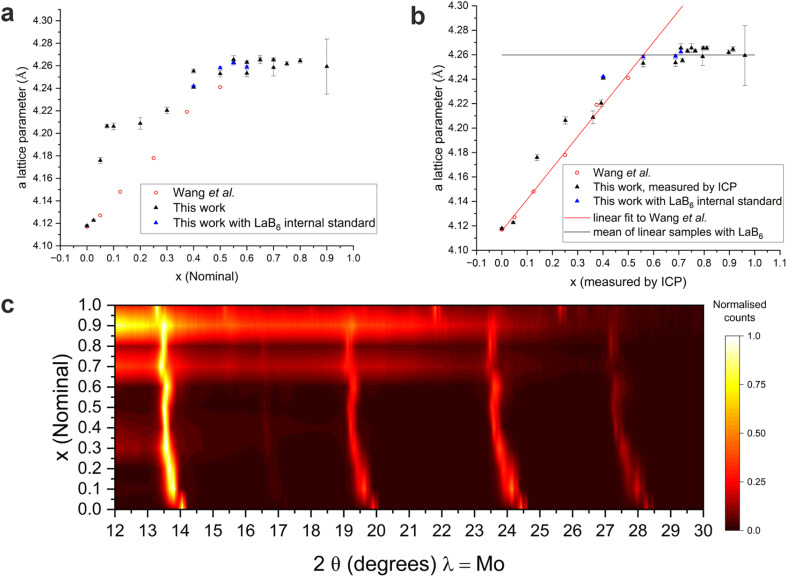
Powder X-ray diffraction of the BaY_*x*_Sn_1−*x*_O_3−*x*/2_ solid solution (a) plot of unit cell parameter *vs.* nominal Y content (*x*) in BaY_*x*_Sn_1−*x*_O_3−*x*/2_ samples prepared using the described high throughput ceramic synthesis route (black and blue data points) and literature values^24^ (red data points); (b) plot of unit cell parameters *vs.* Y content measured by ICP (black and blue data points) overlaid with literature values. The red line indicates the linear fit to literature values, and the black line is the mean value of the last three points measured with LaB_6_, equal to 4.260(14) Å, the two lines intersect at *x* = 0.56, indicating the upper limit of the solid solution. (c) PXRD data from samples annealed at 1400 °C demonstrating peak shift between nominal *x* values between 0 and 1, in steps of 0.1. The colourmap represents the relative intensity of the PXRD data.

The next phase of the work was the exploration of the Ba–Y–Sn–O system using suspensions of BaCO_3_, SnO_2_ and Y_2_(C_2_O_4_)_3_ (yttrium oxalate). Yttrium oxalate was used after initial trials with Y_2_O_3_ showed that the suspensions thickened during storage and became unworkable after a few days. The exploration generated data for 48 compositions at four temperatures (192 individual pellets) in 3 weeks; the researcher was engaged in other tasks during this period. This initial set of trials did not locate any previously unknown phases, but did reproduce the BaY_0.5_Sn_0.5_O_2.75_ quaternary phase reported by Wang *et al.*^24^ To further test the described protocol the quaternary solid solution BaY_*x*_Sn_1−*x*_O_3−*x*/2_ was explored in more detail, in particular to investigate whether *x* = 0.5 was indeed the upper limit of solid solution as is reported. To do this only required two suspensions: an equimolar mixture of BaCO_3_ + SnO_2_ and an equimolar mixture of BaCO_3_ + Y_2_(C_2_O_4_)_3_. Four sets of 10 discs of compositions of BaY_*x*_Sn_1−*x*_O_3−*x*/2_ with *x* = 0 and 0.5 ≤ *x* ≤ 0.95, were produced using the workflow and calcined at temperatures of 1200 °C, 1300 °C, 1400 °C and 1500 °C (one set of discs for each temperature, making a total of 40 samples). X-ray diffraction data from 2*θ* = 10°–52° (*d* = 4.0 to 0.8 Å) were collected and analysed using TOPAS-academic version 7 software.^23^ All of the samples showed a cubic BaSnO_3_ like phase. The unit cell sizes for this cubic phase are shown in Fig. 4 for a set of samples calcined to 1400 °C for 8 hours. The unit cell parameters that we present here (Fig. 4b) are reassuringly similar to the data published by Wang *et al.*^24^ for *x* ≤ 0.5 when plotted against the composition values measured through ICP analysis of the freeze dried mixtures, with a precise match for BaSnO_3_ (*x* = 0) giving *a* = 4.11762(9) Å compared to literature value of 4.117 Å.

The data show the expected increase in unit cell size with increasing *x* up to *x* ≈ 0.56, where the lattice parameter *a* = 4.2582(11) Å for the sample with measured *x* = 0.56, consistent with Vegard's law, which reaches a plateau at *x* ≈ 0.6. Previously, BaY_*x*_Sn_1−*x*_O_3−*x*/2_ phases with *x* ≤ 0.5, with a maximum lattice parameter for *x* = 0.5 of *a* = 4.241 Å, were reported from a sol–gel route with serial synthesis and characterisation of individual samples.^12^ Here we extend the solid solution beyond the limit previously reported, identifying the composition BaY_0.56_Sn_0.44_O_2.72_ as the upper limit of the solid solution limit of the solid (Fig. 4b). This demonstrates that the high-throughput workflow that enables rapid access to a broad range of compositions can reproduce quaternary samples and results produced individually. Removing the samples from the resin and collecting serial diffraction data incorporating a LaB_6_ internal standard (Fig. 4b and c) validated the results from the screening X-ray diffraction and the extension of the solid solution to *x* = 0.56, demonstrating the reliability of the workflow in identifying new compositions based on diffraction from arrays.

### 2.3 Exploration of the Nb–Al–P–O phase space

**Fig. 5:**
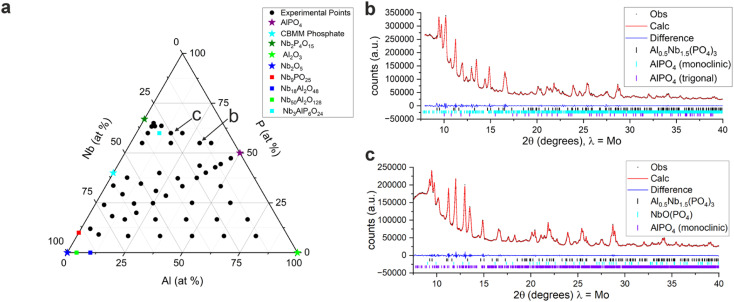
Exploration of the Nb–Al–P–O composition diagram (a) samples prepared in the Nb–Al–P–O composition diagram from the five reagents (represented as stars). Elements are assumed to be in their usual oxidation states (Al^3+^, Nb^5+^, P^5+^) and combined with the stoichiometric amount of oxygen. Square points represent the known compositions in the Nb–Al–P–O phase field; (b and c) Pawley fits to PXRD data from samples made by this route, calcined at 1000 °C, showing the Nb_3_AlP_6_O_24_ quaternary phase^25^ plus AlPO_4_ and NbOPO_4_, compositions of the samples shown are indicated in panel a.

The capability of this workflow was extended further in an exploration of the Nb–Al–P–O system. It is possible to cover over 80% of the chemical space (Fig. 5a) using the starting materials Nb_2_O_5_, Al_2_O_3_, AlPO_4_, Nb_2_P_4_O_15_, and a commercial niobium phosphate (CBMM, Brazil) with a P/Nb ratio of 0.65. The remaining 20% of the phase diagram is not accessible by this method as it requires the use of P_2_O_5_ which is water soluble. 149 individual samples spanning the forty-six compositions within the Nb–Al–P–O phase field shown in Fig. 5a were prepared using appropriate mixtures of the starting materials, calcined at four temperatures (1000 °C, 1100 °C, 1200 °C or 1300 °C) and characterised by X-ray diffraction in a three week period; the researcher was engaged in other tasks during this period.

The known ternary compositions Nb_50_Al_2_O_128_, Nb_18_Al_2_O_48_ and Nb_9_PO_25_, and the Nb_3_AlP_6_O_24_ quaternary (Fig. 5b) reported by Zhao *et al.*^25^ were detected demonstrating that the raw materials do react when processed as described to replicate outcomes previously obtained by conventional solid state processing, affording oxides, phosphates and phosphate oxides with complex structures.

### 2.4 Output rate

The work on the Nb–Al–P–O system generated and characterised 46 compositions calcined at four temperatures producing diffraction data for 149 discs in three weeks. The exploration of the Ba–Y–Sn–O system generated data for 48 compositions at four temperatures (192 individual pellets) in 3 weeks. Both these sample sets were generated by researchers new to the process and included time for data processing and experimental planning. Estimated output for the conventional solid-state synthesis process (hand grind, press, calcine, analyse) is between 10–20 compositions per week for an undergraduate researcher, dependent upon how many re-grinding cycles are needed and how many temperatures have to be addressed. In contrast, the high-throughput method described here requires only a single mixing cycle and, provided enough raw material suspension is available, the added time to prepare an extra set of samples for another temperature or other process condition is negligible – it merely requires dispensing of an extra sample set from the mixed suspensions and transfer of an extra tray between processes.

The Eppendorf liquid handling system has space to generate 240 samples (10 locations each holding two trays with 12 samples in each) from one set of four suspension starting materials in each run and could deliver two runs per day. This is a larger number of samples than could be realistically measured on a lab-based X-ray diffractometer per day, even if the instrument were dedicated to the workflow. Within our current equipment limitations, this would be 4 trays per day (48 samples per day), which could be increased by, for example, increasing the number of individual samples per tray (*i.e.*, reducing the diameter of the wells). A sensible approach would be to prepare the sample sets at the rate at which the X-ray diffractometer can realistically measure (*i.e.*, 48 per day in our case), so there is a continuous flow of samples through the workflow, with each stage always having a sample set in process, and the ability for results to feedback and influence the experimental design of new samples sets in a timely manner.

The total process time from wet milling to data analysis is currently 8 days, with overnight processing for freezing, drying, calcination (48 hours), resin curing and X-ray diffraction. Optimisation of the freezing method and a rapid curing resin could reduce the total process time to four working days allowing faster feedback of results into the workflow and permitting a repeatable one week cycle including some time for data analysis. Total process time will, of course, depend on the chemical systems being explored and local facilities.

With the capability of generating large numbers of samples, sample tracking may become a serious issue. At present the location of the samples in the sets of 12 is indicated by cutting off one corner of the PET trays, marking the location of sample 1 in each set (Fig. S1†). This mark is maintained by the use of a small ceramic crucible during calcination and filling in one corner of the 3D printed PLA frame as shown in Fig. 2c, and in more detail in Fig. S3.† This avoids problems due to rotation of the sample sets and further tracking of the samples relies on careful manual record keeping, including photographing marked sample trays, which is adequate for our current diffraction limited rate of 4 trays of samples per day. High temperature capable barcodes/QR-codes are available (Lintec Europe, HP12 3SL, UK) that can be applied onto small ceramic plates, possibly made from alumina substrate for example as supplied by Almath Crucibles, (CB8 9NE, UK). These could be added to an extra well in the PET sample trays and transferred through the workflow along with the relevant sample set, allowing tracking of large numbers of samples.

## 3 Conclusions

The workflow presented here can be used to screen a wide variety of inorganic oxide and related systems for unknown phases, demonstrated here with the observation of a range of quaternaries, including a previously unreported composition. The workflow begins to offer a high throughput approach to surveying increasingly complex systems with the potential for further development to increase the throughput rate and add additional automation according to the requirements of the particular research task. The aim is to remove lengthy manual steps while avoiding investing considerable resources in engineering every single step, when there are steps that are better and more flexibly done manually in a research environment. The data generated are comparable to data that are obtained from conventional methods, which demonstrates the applicability of the workflow for the identification of new phases. The workflow gives access to a range of chemistries and reaction environments matching those in traditional solid-state synthesis, and affords samples in a form factor compatible with a range of measurements.

## Data availability

Underlying data collected as part of this work are available at the University of Liverpool data repository at https://datacat.liverpool.ac.uk/id/eprint/2478.

## Author contributions

C. J. H. and M. J. R. developed the project direction. C. J. H. developed the described procedures. M. P. S. and L. L. A. utilised the developed procedure to perform the Ba–Y–Sn–O and Nb–Al–P–O syntheses, respectively. L. M. D., T. D. M., M. W. G., J. B. C., and M. J. R. advised on procedure development. C. M. C. and L. M. D. performed refinements against powder diffraction data. C. J. H. and T. D. M. wrote the initial draft of the manuscript. All authors contributed to discussion and editing of the manuscript. M. J. R. directed the research.

## Conflicts of interest

There are no conflicts to declare.

## Supplementary Material

SC-015-D3SC05688K-s001
