# Supplementary material for: A high throughput synthetic workflow for solid state synthesis of oxides
Source: Chem Sci. 2024 Jan 2;15(7):2640–7. doi: 10.1039/d3sc05688k (PMC10866347; doi:10.1039/d3sc05688k)
Supplement: SC-015-D3SC05688K-s001 [file SC-015-D3SC05688K-s001.pdf]

This journal is © The Royal Society of Chemistry 2024

A High Throughput Synthetic Workflow For Solid State Reactions

Christopher J. Hampson, Moli P. Smith, Luca L. Arciero, Christopher M. Collins, Luke M. Daniels, Troy D. Manning, Michael W. Gaultois, John B. Claridge, Matthew J. Rosseinsky

Department of Chemistry, University of Liverpool, Materials Innovation Factory, 51 Oxford Street, Liverpool, L7 3NY

Supplementary Information

Methods

Sample tracking and location

As the array of samples is symmetrical, in order to retain the identity of individual discs within the array the PET trays and the PLA frames have one corner cut off, indicating the position of sample 1 in the array.

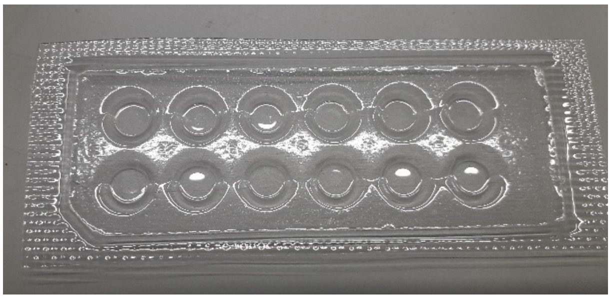

Fig. S 1. Vacuum formed PET tray with cut off corner indicating the position of Sample #1 in the sample set.

Isopressing

The empty isopressing tool, used in Step 5 of the workflow, is shown in main text Fig. 2b. The complete assembly including the sample discs on their PET tray, and the surrounding evacuated nylon bag is illustrated in the schematic in Fig. S 2.

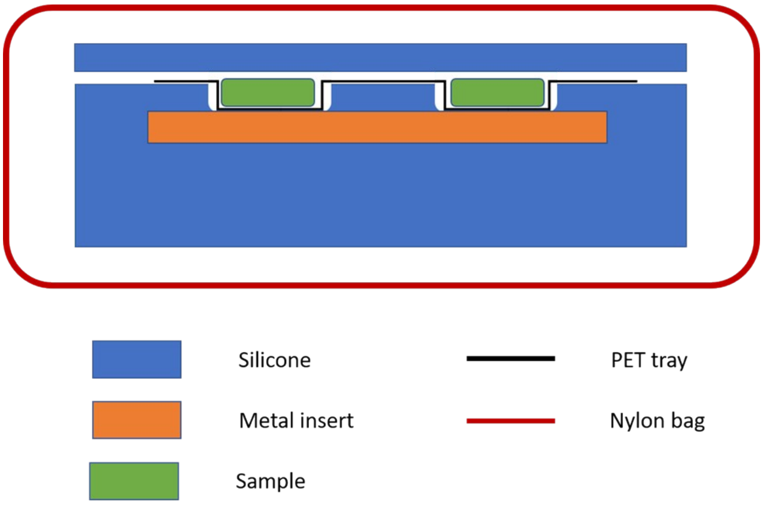

Fig. S 2 Schematic representation of the sample preparation for iso-pressing

The metal insert (3mm brass sheet) keeps the bottom of the samples flat which is important for the X-Ray Diffraction analysis. The lower silicone part is made by casting a two-part room temperature curing silicone resin (DWR Plastics, TF10 7TP, UK Silicone Moulding Rubber Shore Hardness: A28) into a mould made using

a PET sample tray and the brass insert, arranged so that the tray can be removed but the brass insert is retained within the silicone. The upper silicone layer can be a 2mm silicone sheet or a cast piece.

X-Ray Diffraction

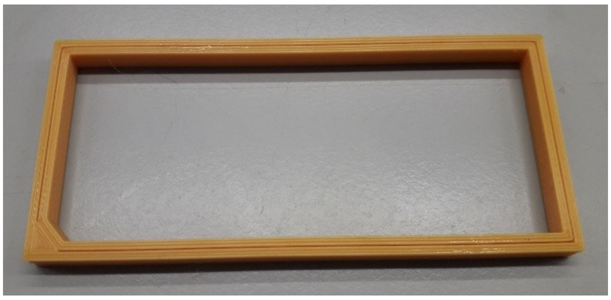

Fig. S 3 3D printed PLA frame with cut off corner to maintain the position of Sample #1 in the sample set.

Step 7 of the workflow (main text, Fig 1a) presents sample sets to an X-ray diffractometer. The way in which the 3D printed sample holder shown in main text Fig. 2d holds the samples parallel to the table of the X-Ray Diffractometer is illustrated in the schematic below.

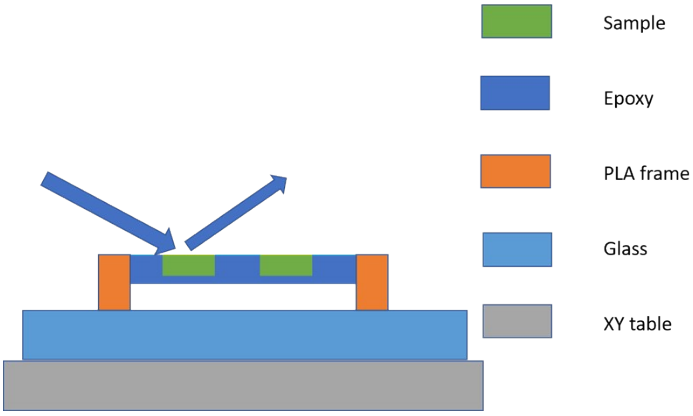

Fig. S 4 Schematic representation of a sample set embedded in epoxy mounted on a XY table of a diffractometer.

The glass plate (6mm soda lime glass) was added to eliminate small peaks due to the stainless steel XY table which were observed in some early experiments.

Bending of samples embedded in epoxy resin

The asymmetric filling of the frames which is required in this step can lead to bending as the resin completes its curing. In early experiments the bending was evident after 24 hours curing and increased with time. Thirty days after casting, early frames showed up to 0.5mm bend leaving the top surface convex and defeating the aim of presenting a flat sample array to the diffractometer.

Two solutions to this problem were identified. Curved blocks could be straightened by putting them on a flat metal plate and under a flat metal weight (> 500g) in an oven at 125C for 1 hour and then cooling with the plate and weight still in place. To avoid having to do this extra step, trials were carried out in which the hardener to resin ratio was varied and the bowing of the frames after the resin had been cured was measured using an engineer's dial gauge. Using Mouldcraft CLR resin, which had a recommended hardener to resin ratio of 0.5:1, the trials covered ratios from 0.2:1 to 0.5:1. The mixture tested at 0.2 :1 hardener / resin ratio remained viscous and tacky, and was not able to be measured. At the standard ratio of 0.5:1 the

frame bowed by almost 1mm after 22 days curing but this could be reduced to <0.2mm by using a hardener to resin ratio of 0.3 – 0.4.

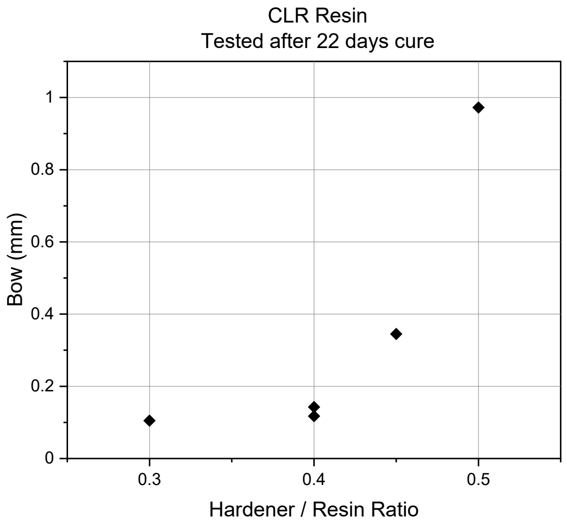

Fig. S 5 Bending of CLR resin in PLA frames versus hardener / resin ratio

Results

Dispensing of $CaCO_3$

To test the effectiveness of the stirrer system at preventing settling of the suspension 1 cm^3 aliquots were dispensed from a suspension of calcium carbonate into glass vials after periods of 1, 3, 10 and 30 minutes. The solids contents of the dispensed suspensions were determined from the loss in weight after oven drying at 80 °C. Fig. S6 shows the weight of liquid dispensed and the weight of solids obtained from it. The consistency of both liquid and solid weights demonstrates that settling has been prevented.

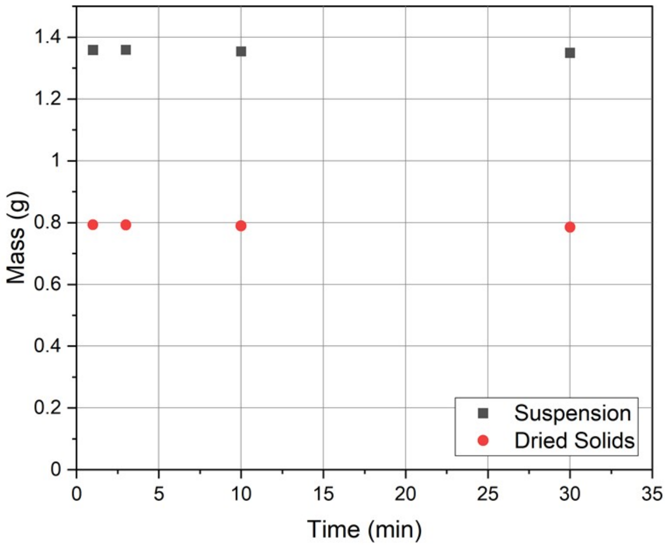

Fig. S 6 Dispensing of calcium carbonate suspension. The standard error of the solids weight is only 0.2% of the mean value. [SE = sd/✓N]

The deviations observed from the expected compositions of the samples described in the Ba-Y-Sn-O phase space were all in the direction of positive x (Y rich) and ranged from $\Delta x = 0$ to $\Delta x = 0.3$ with an average deviation of $\Delta x = 0.1$. For the purpose of screening the composition space these deviations are adequate

and allow the identification of regions of interest, though further work is required to optimise the dispensing parameters of the liquid handling system to accommodate the higher viscosity and density of the suspensions compared to most aqueous solutions and reduce these deviations. As in this work, samples of particular interest can be extracted from the resin block for chemical analysis.
